# Safety and Efficacy of Stromal Vascular Fraction Enriched Fat Grafting Therapy for Vulvar Lichen Sclerosus

**DOI:** 10.7759/cureus.7096

**Published:** 2020-02-25

**Authors:** Juan Monreal

**Affiliations:** 1 Plastic Surgery, Hospital La Luz, Madrid, ESP

**Keywords:** vulvar dermatoses, lichen sclerosus, lichen simplex chronicus, lichen planus, stromal vascular fraction, fat grafting, adipose derived stem cells

## Abstract

Background

Lichen sclerosus is an inflammatory dermatosis of unknown etiology which currently has no cure. Most treatment guidelines recommend the use of ultrapotent topical corticosteroids. However, the relapse rate is usually high. Through a retrospective study we evaluated the efficacy and safety of stromal vascular fraction of adipose tissue as therapy for lichen sclerosus.

Material and methods

For this retrospective review, we obtained data on patients with vulvar lichen sclerosus treated with autologous fat grafting enriched with adipose derived stromal vascular fraction cells. Data collected through a modified vulvo-vaginal symptoms questionnaire were analyzed before treatment, six months and 24 months after treatment. The 19 items questionnaire was subdivided in four categories: symptoms, signs, social functioning and sexual functioning. Global scores and partial scores for each category were analyzed using paired t-test. For all statistical analyses, a value of *p ≤* 0.05 was considered statistically significant. All data are presented as mean ± SD.

Results

Thirty nine patients were included in the study. Thirty seven patients (94.87%) experienced a significant decrease in global score at six months and 24 months after treatment (p < 0.05). Decrease in scores were also statistically significant between pretreatment and 24 months after treatment for each of the four questionnaire categories - symptoms, signs, social functioning and sexual functioning (p < 0.05).

Conclusions

This retrospective study showed that the use of autologous fat grafting enriched with adipose derived stromal vascular fraction is safe and leads to significant and long lasting improvement in patients with vulvar lichen sclerosus.

## Introduction

Vulvar dermatoses is the term which groups different diseases such as lichen sclerosus, lichen simplex chronicus and lichen planus. Lichen sclerosus is an inflammatory disease that primarily affects the skin of female external genitalia and glans and foreskin of male patients [1]. Approximately 6% of these patients have extra-genital involvement and between 85% -98% have anogenital involvement [2]. It is the second leading cause of non-neoplastic vulvar disease, with an estimated prevalence of 1:300-1:1000. It is considerably more common in women than in men at a ratio of 10: 1 and, although it may appear throughout life, is more frequent in perimenopausal women and girls. Children, especially prepubertal girls, may be affected representing 7-10% of all cases.

Lichen sclerosus has an unknown etiology but there is evidence to suggest that autoimmune mechanisms are involved in it's pathogenesis [3]. Some studies have observed high rates of autoimmune diseases in patients with lichen sclerosus and higher levels of detection of autoantibodies. It has been shown that patients with vulvar lichen sclerosus have an increased risk of developing vulvar squamous cell carcinoma, this risk being estimated at 5% [4]. Although there are no studies showing the contrary, it is believed that early and effective treatment would reduce this risk. Men with genital lichen sclerosus also have increased risk of developing squamous cell carcinoma of the penis.

Clinically, lichen sclerosus usually begins as erythematous papules or white patches that slowly coalesce to form larger plaques. Extragenital lesions are usually asymptomatic or poorly symptomatic, but genital lesions usually present with a varied combination of intense itching, burning or pain. Genital lesions may become extremely devastating, particularly in women, producing agglutination, loss of the labia minora and clitoral hood, vaginal introitus stenosis or involvement of the urethral meatus. Peri-anal involvement is also especially common in women, with the presence of fissures, hyperkeratosis, sclerotic plaques and their corresponding symptoms. It is easy to understand that the quality of life of patients with lichen sclerosus, especially women, is extremely impaired.

The long-term evolution of vulvar lichen sclerosus varies greatly depending on the severity of each case and compliance with treatments. A high percentage of cases often suffer from severe physical dysfunction, notorious deformities and severe deterioration in the quality of life, social relationships and sexual functioning. It is therefore extremely important to conduct an early diagnosis and establish some form of treatment to control symptoms and disease progression. Additionally, the occasional side effects that can be derived from chronic corticosteroids use must be added to the effects of the disease itself.

The purpose of this study is to evaluate the efficacy and safety of a treatment based on fat grafting enriched with adipose derived stromal vascular fraction (SVF) cells as numerous studies conclude there is evidence of anti-inflammatory and immunoregulatory properties of this heterogeneous cell population (properties that can be used to control diseases such as lichen sclerosus). In addition to its effectiveness, the aim of this study is to evaluate how long patient improvement can last.

## Materials and methods

Patients and design

From November 2012 to October 2016, the author performed a total of 90 treatments in 82 patients with genital involvement caused by some of the most common vulvar dermatosis. Sixty two cases corresponded to lichen sclerosus, eight to lichen planus and twelve to lichen simplex chronicus. This is a retrospective study of treated cases that matched inclusion criteria. Inclusion criteria were the presence of vulvar lesions diagnosed as lichen sclerosus, at least twenty four months follow-up after treatment, and have completed at least one modified vulvovaginal symptoms questionnaire (VVSC) before treatment, six months and 24 months after treatment. Male patients with lichen sclerosus and all female patients with lichen planus and lichen simplex chronicus were excluded from the study due to the insufficient number of cases. Lichen sclerosus patients with less than twenty four months follow up, patients who were given a second treatment and those from whom was not possible to get any of the postoperative health questionnaires were also excluded from the study. Consequently, the present study only analyzes thirty nine female patients with vulvar lichen sclerosus in which a single treatment was performed.

Age of the disease before treatment was impossible to determine in most patients due to the lack of objective data, or absence of relevant clinical documentation. The shortest estimated time of evolution from the correct diagnosis to treatment was two years, with seventeen years being the longest time recorded before treatment. All patients were diagnosed in other institutions, by biopsy in 20% and clinically in the remaining 80%. Before treatment, suspicious neoplastic or pre-neoplastic lesions was discarded. All patients that were using topical clobetasol propionate ointment 0.05% or tacrolimus ointment 0.1% prior to treatment were advised not to use it without notifying. The objective of this measure was to avoid the influence of these topical treatments in the correct monitoring and evaluation of cell behavior.

Ethics

All patients were informed about the nature of the treatment and the degree of improvement expected in each case. All patients were told that the aim of treatment was improving quality of life by improving signs and symptoms of the disease without the need of topical medications, and thus allowing and enhancing regeneration of damaged tissues at the same time. All patients with anatomical sequelae (loss and agglutination of the labia minora, midline fusion with loss of the clitoral hood, stenosis of vaginal introitus, etc) were told that the treatment won’t revert any of them.

Questionnaires and data collection

Clinical data, physical examination, photographs of the external genital area and a questionnaire based on the modified Spanish version of the vulvovaginal symptoms questionnaire (VVSQ) were obtained from each patient (Table 1). The original VVSQ contains nineteen ungrouped items with a simple boolean answer (Yes/No). The modification of this questionnaire consisted on expanding the range of original answers to eleven (0 expressing total absence of problems to 10 expressing the greater severity of the problem) and reorganizing the items of the questionnaire into four categories: symptoms, signs, social functioning and sexual functioning. Thus, for each questionnaire, a global score (score range 0-190) and partial scores for each category were obtained (symptoms score range 0-40, signs score range 0-40, social functioning range 0-80, sexual functioning score range 0-30). After treatment, new questionnaires were completed, one for each follow up visit. For the final analysis preoperative questionnaires and those obtained in the 6th and 24th month after treatment were selected.

**Table 1:** Vulvovaginal symptoms questionnaires. Vulvovaginal symptoms questionnaires (VVSQ). Modified and translated questionnaire with questions reorganized in four categories (left). Original questionnaire (right). These questions were score on a scale of 0-10 with 0 expressing total absence of problems to 10 expressing the greater severity of the problem.

Symptoms (in spanish)	Symptoms
Picores vulvares	Is your vulva itching?
Quemazón vulvar	Is your vulva burning or stinging?
Dolor vulvar	Is your vulva hurting?
Dolor en las relaciones sexuales	Are your vulvar symptoms causing pain during sexual activity?
- SIGNS	- SIGNS
Irritación de la piel vulvar	Is your vulva irritated?
Sensación de sequedad	Is your vulva dry?
Presencia de flujo	Is there discharge from your vulva or vagina?
Presencia de olor	Is there odor from your vulva or vagina?
- SOCIAL FUNCTIONING	- SOCIAL FUNCTIONING
¿Está preocupada por sus síntomas?	Do you worry about your vulvar symptoms? (for example, that it will spread, get worse, scar, etc.)
¿Esté preocupada por su aspecto?	How is the appearance of your vulva?
¿Siente frustración por su situación?	Are you frustrated about your vulvar symptoms?
¿Siente vergüenza por su situación?	Are you embarrassed about your vulvar symptoms?
¿Afecta su situación al trato con otros?	Do your vulvar symptoms affect your interactions with others?
¿Rechaza la compañía de otros?	Do your vulvar symptoms affect your desire to be with people?
¿Su afectividad se ha visto alterada?	Do your vulvar symptoms make it hard to show affection?
¿Se han visto alteradas sus actividades diarias?.	Do your vulvar symptoms affect your daily activities?
- SEXUAL FUNCTIONING	- SEXUAL FUNCTIONING
¿Se ha visto alterado el modo en el que mantiene relaciones sexuales?	Do your vulvar symptoms affect on your sexual relationships?
¿Tiene mayor sequedad durante las relaciones sexuales?	Do your vulvar symptoms cause dryness during sexual activity?
¿Ha tenido sangrado durante las relaciones sexuales?	Do your vulvar symptoms cause bleeding during sexual activity?

Table 2 shows the descriptive statistics of the study. The mean age was 45.79 ± 13.64 (range from 13 to 69 years). Kolmogorov-Smirnov test was used to determine that sampled data had a normal distribution. Paired sample t test was used to find the differences in global VVSQ scores and differences in scores for each category (symptoms, signs, social and sexual), prior to surgery, six months after the surgery and 24 months after surgery. Finally, we analyzed whether there were statistical significant differences between the global scores obtained at sixth and 24th month after treatment, which would give relevant information about duration of results. The level of significance was set at 5% (p < 0.05) for all tests and PSPP software, version 1.2.0-2 (https://www.gnu.org/software/pspp/) was used to perform all statistical analysis. All results are expressed as mean, +/- standard deviation.

**Table 2:** Descriptive statistics

	Valid cases = 39; case(s) with missing value(s) = 0.						
Variable	N	Mean	Std Dev.	Curtosis	Asymmetry	Lower	Upper
Age	39	45.79	13.64	-.25	-.61	13.00	69.00
Symptoms Score Pre	39	21.18	9.51	-.81	-.01	5.00	40.00
Signs Score Pre	39	16.90	8.71	.19	.39	2.00	40.00
Social Score Pre	39	39.85	20.09	-.82	.45	7.00	80.00
Sexual Score Pre	39	15.64	10.58	-1.30	-.11	.00	30.00
Global Score Pre	39	93.56	33.97	-.23	.42	32.00	171.00

Surgical technique

Adipose Tissue Harvest

All treatments were performed under local anesthesia with sedation. Adipose tissue was harvested from abdomen and/or inner thighs. The procedure for obtaining adipose tissue for fat grafting was described by the author elsewhere and is briefly described [5]. First donor adipose tissue is infiltrated using a tumescent solution consisting of lactated Ringer's solution 1000 ml, Lidocaine 500 mg and epinephrine 1: 1000, 1 mgr. Harvesting of adipose tissue was performed using 10 ml syringes attached to multi-holed 2,3 and 4 mm cannulas. Harvested fat was washed with bicarbonate Ringer's solution (lactated Ringer's solution 1000 ml plus Bicarbonate 10M, 10 ml) and decanted for 20 to 30 minutes.

Stromal Vascular Fraction Isolation

The mean weight of processed lipoaspirate obtained was 70 gr (range 55 to 80 gr), of which approximately 50 to 60 gr were used to obtain SVF and the remainder was used as graft. SVF was obtained by enzymatic digestion of lipoaspirate using a collagenase solution, with a final concentration of 0.15 PZ / ml, for 45 minutes at 37°C on an orbital shaker (Collagenase NB6 GMP, Nordmark Arzneimittel GmbH & Co. KG, Germany). The digested lipoaspirate was centrifuged for 10 minutes at 800g to obtain the pellet containing SVF cells. The pellet obtained was resuspended using lactated Ringer's, filtered using a 100 μm microfilter and resuspended - centrifuged three times to remove any residual collagenase activity. The final SVF cell suspension ranged from 5 to 6 ml. For each patient a cell count using Adam-MC device (NanoEnTek, Seoul, Korea) was performed, obtaining a mean yield of 643,990 cells/gr of adipose tissue and an mean viability of 87%.

Mixture and Infiltration

Approximately 15-20 ml of adipose tissue reserved for fat grafting was mixed with 3.3 to 4.3 ml of the SVF cell suspension and used to infiltrate subdermis and subcutaneous tissue. Approximately 1.5 ml of the SVF cell suspension was used for intradermal injection. Additionally 0.2-0.3 ml of the SVF cell suspension was processed for cell counting. The infiltration was performed in vulvar, perineal and/or perianal areas according to clinical manifestations and physical exam.

Postoperative course

All patients were discharged without incident and followed at regular intervals of 1.5 months, 3 months, 6 months, 12 months, 18 months and 24 months. In each visit, each patient completed a new VVSQ as part of follow-up. All patients experienced mild discomfort and swelling in the genital area that resolved within a maximum period of six days. Discomfort and inflammatory manifestations of donor adipose tissue area lasted 6 weeks. No patient experienced complications or side effects throughout the duration of the study. Patients that were using Clobetasol propionate ointment 0.05% experienced some worsening of their symptoms after treatment. This crisis slowly disappeared over the first three weeks and was extremely tolerable after the sixth week. Patients were allowed to use local lubricants and moisturizers if needed.

## Results

**Figure 1:**
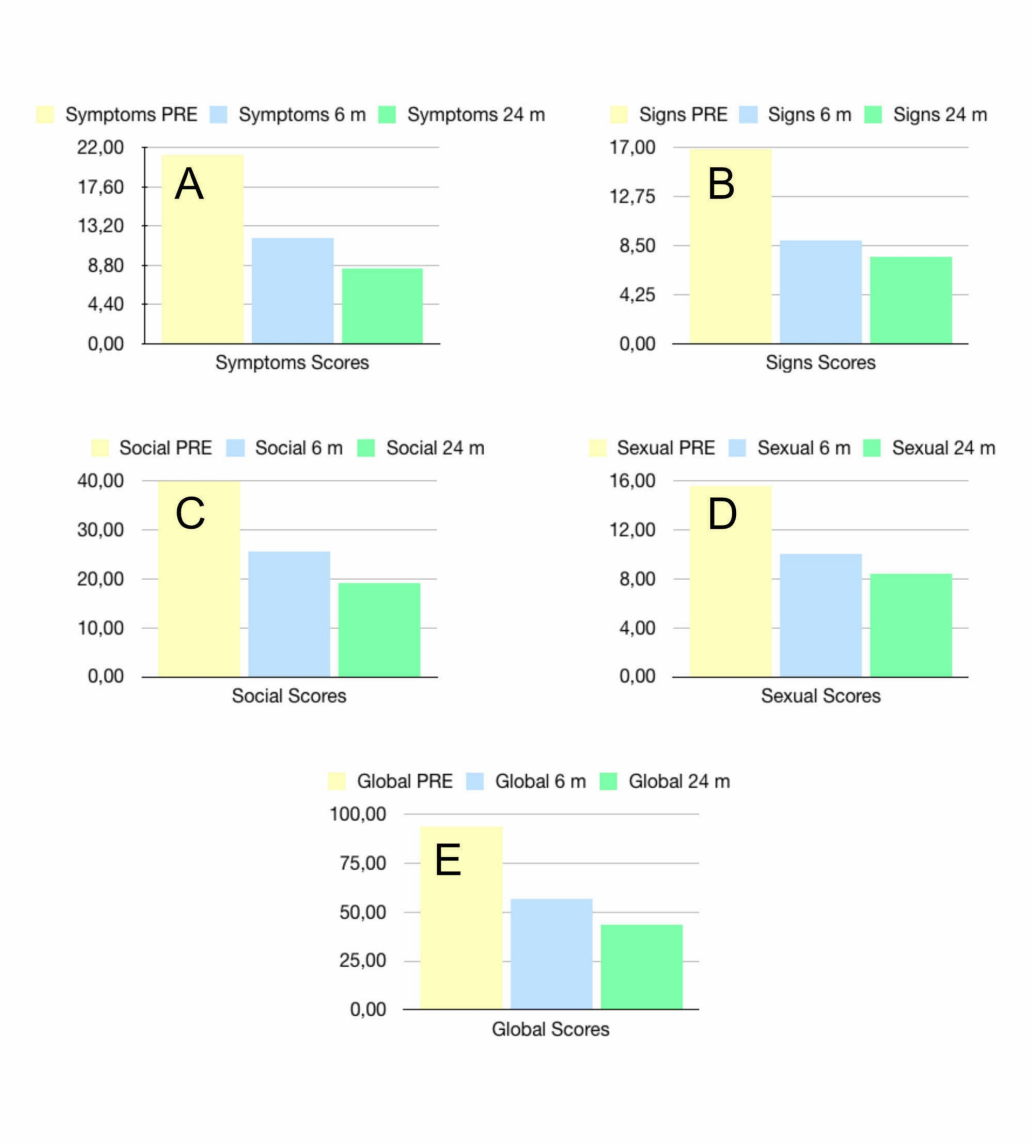
The mean scores of VVSQ reported by patients at pretreatment, six and twenty four-month follow-ups. A) Symptoms scores B) Signs scores C) Social functioning scores D) Sexual functioning scores E) Global scores

Of the thirty nine patients included in the study, thirty seven patients (94.87%) experienced positive changes in global scores at six and 24 months. Only two patients (5.12%) experienced negative changes and in both cases the patients confessed having used Clobetasol sometime after treatment. The test results showed a global score at 24 months significantly lower than before treatment (43.59 ± 28.23 vs 93.56 ± 33.97, p<0,05). In the categories of symptoms, signs, social functioning and sexual functioning, scores at 24 months (8.46 ± 7.28, 7.51, ± 5.96, 19.18 ± 18.36 and 8.44 ± 7.95 respectively) were significantly lower than those obtained before treatment (21.18 ± 9.51, 16.90 ± 8.71, 39.85 ± 20.09 and 15.64 ± 10.58 respectively; p<0,05). The global scores at 24 months were also significantly lower than those obtained at six months (43.59 ± 28.23 vs 56.59 ± 28.70; p<0,05). Symptom scores and social functioning scores at 24 months (8.46 ± 7.28 and 19.18 ± 18.36 respectively) were also statistically lower than those obtained at six months (11.92 ± 6.83 and 25.64 ± 17.97 respectively; p<0,05). Signs scores and sexual functioning scores were lower at 24 months (7.51 ± 5.96 and 8.97 ± 6.01 respectively) than at six months (10.05 ± 8.24 and 10.05 ± 8.24 respectively) but the difference was not statistically significant. This finding correlates with the subjective patient feeling that improvement was maintained or discretely improved after the first six months. Table 3 summarizes all the scores and Figure 1 shows the results as bar charts. Refer to appendices to view complete paired t-Test tables.

**Table 3:** Summary of scores obtained from VVSQ SD= standard deviation

	Symptoms		Signs		Social Functioning		Sexual Functioning		Global	
	Score	SD	Score	SD	Score	SD	Score	SD	Score	SD
Preoperative	21.18	9.51	16.90	8.71	39.85	20.90	15.64	10.58	93.56	33.97
6 months	11.92	6.83	8.97	6.01	25.64	17.97	10.05	8.24	56.59	28.70
24 months	8.46	7.28	7.51	5.96	19.18	18.36	8.44	7.95	43.59	28.23

## Discussion

According to the latest clinical guidelines on the management of patients with lichen sclerosus, there are two main lines of treatment [6-7]. The recommended treatment is the application of ultrapotent topical corticosteroid like clobetasol propionate ointment 0.05%. The second line of treatment involves topical application of calcineurin inhibitors, particularly the application of tacrolimus 0.1%. Although it can be used as a first option, this second line of treatment is usually reserved for cases where the use of topical corticosteroids is not well tolerated or do not adequately control the disease.

Additional treatments have been described in order to control the symptoms and signs of lichen sclerosus. Many of them have been described as alternatives in case of failure or lack of tolerance with the aforementioned treatments. Some of the most common reported alternatives are intralesional corticosteroid (triamcinolone), topical application of testosterone, estrogens or progesterone, retinoid creams (tretinoin, acid 13-cis retinoic acid and retinaldehyde), UV phototherapy, photodynamic therapy, cyclosporin, methotrexate, hydroxychloroquine, antibiotics, etc [8-12]. It should be emphasized that treatment with an ultrapotent corticosteroid can improve but does not cure lichen sclerosus, and it appears that in patients where a remission was achieved, this was only temporary [13].

Although the potential of adipose tissue as regenerative therapy is well known for some time, Zuk et al demonstrated that this potential is probably provided by its high content of adult stem cells [14]. Since then, adipose tissue derived stem cells have been extensively studied because of their regenerative capacity, and immunoregulatory and anti-inflammatory properties [15,16]. Several of these studies support the concept that these capabilities probably reside in the relationship of adipose derived stem cells with other cell lines that live with them in adipose tissue, all of which are given the name of stromal vascular fraction.

**Figure 2:**
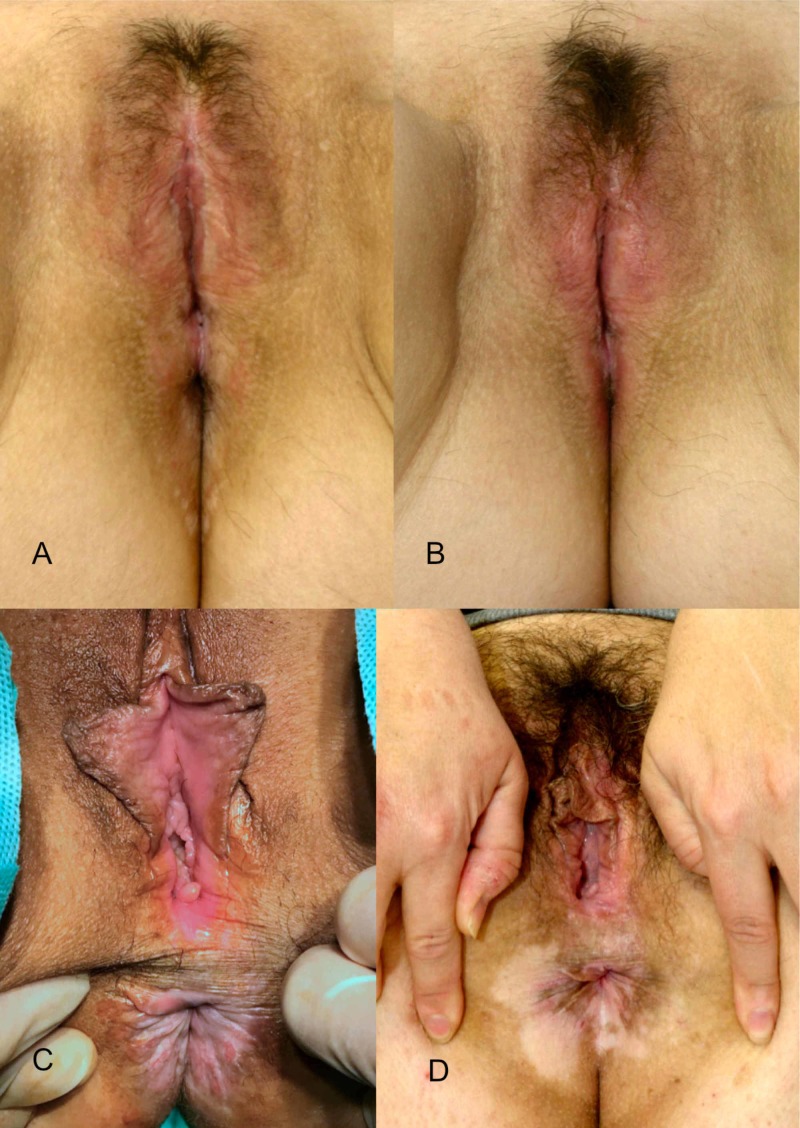
Preoperative and postoperative results after 24 months and one single treatment. A-C.- Preoperative images of two patients affected by lichen sclerosus  B-D.- Postoperative results after 24 months.

Although the adipose tissue obtained from a liposuction, can be used as regenerative therapy, cell concentrations inside a simple fat graft are not large enough to have a significant clinical impact on certain diseases where immunomodulation is crucial. To overcome this limitation, isolation the SVF from adipose tissue by different methods allows the use of fat grafting with a higher and clinically more relevant concentration of regenerative cells. Figure 2 shows preoperative and postoperative results at 24 months.

The use of PRP (Platelet Rich Plasma) alone or associated with fat grafting as an alternative treatment for lichen sclerosus has been investigated previously. Goldstein et al describe the effectiveness of two intradermal and subdermal applications of PRP, separated six weeks apart, in 15 patients with vulvar lichen sclerosus [17]. Similarly, Behnia-Willison et al describe their positive experience with the use of multiple injections of PRP in 28 patients who were almost all postmenopausal, and affected by vulvar lichen sclerosus, using Pelvic Floor Questionnaire to validate the results [18]. Casabona et al summarize their positive experience in 12 patients using fat grafts followed by PRP, although they do not use any evaluation questionnaire to validate their results [19]. Newman et al published a series of 100 patients treated with what they call autologous adipose-derived stem cell treatment [20]. Although the number of patients and the results are significant and the results are supported by a questionnaire, only responses from before the procedure as compared to three months after treatment were analyzed. Onesti et al published an essay in eight patients suffering from various vulvar dystrophies of which only five correspond to lichen sclerosus [21]. The treatment consisted of two injections of a suspension of expanded adipose derived stem cells in hyaluronic acid.

Although all the studies mentioned above report good results, none of them uses specific health questionnaires for the vulvovaginal area to validate their results. After an extensive literature research, we believe that this is the first description of the use of SVF enriched fat grafting for vulvar lichen sclerosus using a specific vulvo-vaginal questionnaire to validate the results. This study shows a significant improvement in most patients at six months, improvement that was increased or maintained for at least 24 months. It is interesting to note that the onset of symptomatic improvement in all patients analyzed did not occur until the third to fourth week after treatment and that the initial improvement increased slowly over the following months.

The limitations of this study are derived from a limited number of cases not categorized by severity, by age ranges or times of evolution and due to it's retrospective nature. Future prospective studies are planned to compare pre-treatment and post-treatment histological analysis.

## Conclusions

This study demonstrates that patients suffering from vulvar lichen sclerosus can be treated safely and effectively with SVF enriched fat grafting. The improvement in quality of life and its duration are significant enough to consider this technique as a therapeutic alternative. Clinically, it has been shown that those with apparently shorter evolution time between diagnosis and treatment, and those with less inflammatory and established lesions responded better and faster, but this is a fact that should be investigated further. Prospective studies with more patients and longer follow ups should be performed to validate these results.

## Appendices

**Table 4:** Paired t test used to test if there was a significant difference between pretreatment and post treatment scores at 6 months.

Paired Samples Test								
	Paired Differences							
				95% Confidence Interval of the Difference				
	Mean	Std. Deviation	Std. Error Mean	Lower	Upper	t	df	Sign. (2-tailed)
Symptoms Score Pre - Symptoms Score 6 M	9.26	9.02	1.44	6.33	12.18	6.41	38	.000
Signs Score Pre -Signs Score 6 M	7.92	8.89	1.42	5.04	10.81	5.56	38	.000
Social Score Pre - Social Score 6 M	14.21	12.65	2.03	10.10	18.31	7.01	38	.000
Sexual Score Pre - Sexual Score 6 M	5.59	7.41	1.19	3.19	7.99	4.71	38	.000
GOBAL SCORE PRE - GLOBAL SCORE 6 M	36.97	24.41	3.91	29.06	44.89	9.46	38	.000

**Table 5:** Paired t test used to test if there was a significant difference between pretreatment and post treatment scores at 24 months.

Paired Samples Test								
	Paired Differences							
				95% Confidence Interval of the Difference				
	Mean	Std. Deviation	Std. Error Mean	Lower	Upper	t	df	Sign. (2-tails)
Symptoms Score Pre - Symptoms Score 24 M	12.72	9.48	1.52	9.64	15.79	8.37	38	.000
Signs Score Pre - Signs Score 24 M	9.38	9.50	1.52	6.30	12.46	6.17	38	.000
Social Score Pre - Social Score 24 M	20.67	17.98	2.88	14.84	26.50	7.18	38	.000
Sexual Score Pre - Sexual Score 24 M	7.21	7.35	1.18	4.82	9.59	6.13	38	.000
Global Score Pre - Global Score 24 M	49.97	33.40	5.35	39.15	60.80	9.34	38	.000

**Table 6:** Paired t test used to test if there was a significant difference between post-treatment scores at 6 months and 24 months.

Paired Samples Test								
	Paired Differences							
				95% Confidence Interval of the Difference				
	Mean	Std. Deviation	Std. Error Mean	Lower	Upper	t	df	Sign. (2-tails)
Symptoms Score 6 M - Symptoms Score 24 M	3.46	5.21	.83	1.77	5.15	4.15	38	.000
Signs Score 6 M - Signs Score 24 M	1.46	6.00	.96	-.48	3.41	1.52	38	.137
Social Score 6 M - Social Score 24 M	6.46	11.21	1.80	2.83	10.10	3.60	38	.001
Sexual Score 6 M - Sexual Score 24 M	1.62	5.56	.89	-.19	3.42	1.82	38	.077
Global Score 6 M - Global Score 24 M	13.00	20.82	3.33	6.25	19.75	3.90	38	.000
